# Acute Localized Exanthematous Pustulosis Induced by a Spider Bite

**DOI:** 10.4269/ajtmh.20-0138

**Published:** 2020-09

**Authors:** Maha Lahouel, Sana Mokni, Mohamed Denguezli

**Affiliations:** Department of Dermatology, Farhat Hached Hospital, Sousse, Tunisia

**Figure 1.**
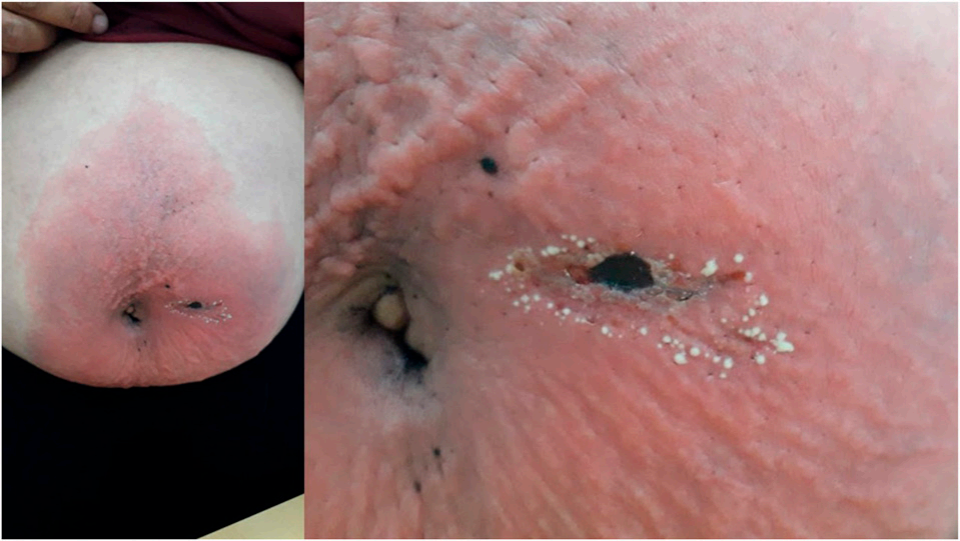
Localized erythema covered with numerous non-follicular pustules centered by a necrotic lesion following a spider bite. This figure appears in color at www.ajtmh.org.

**Figure 2.**
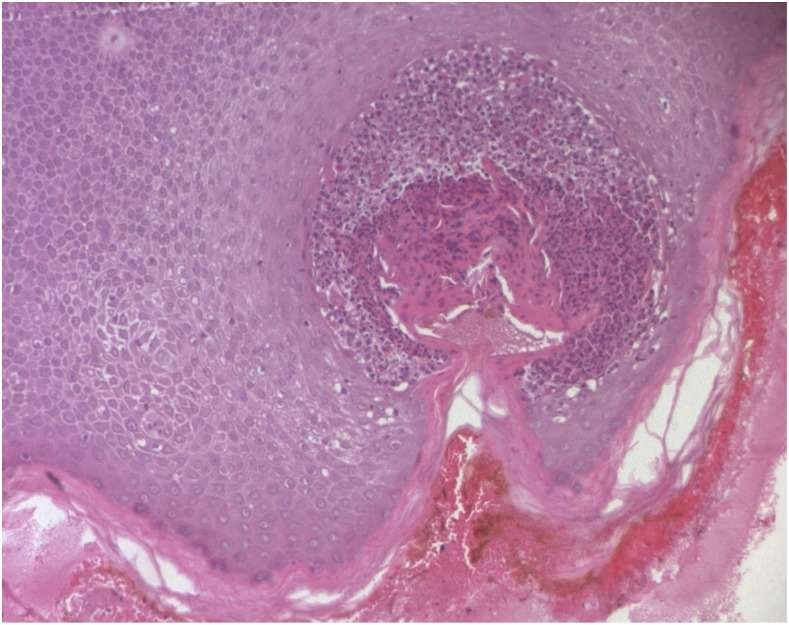
Intraepidermal pustule and mild spongiosis associated with an inflammatory infiltrate (hematoxylin–eosin, ×100). This figure appears in color at www.ajtmh.org.

A 52-year-old woman, of rural origin, with no medical history, was admitted to our hospital for acute outbreak of multiple pustules on an underlying edematous erythema, localized on the abdomen below the navel evolving for 48 hours. The patient denied using of any prescription or over-the-counter medication before the onset of symptoms. An accurate medical history revealed that it had a sudden onset after an accidental spider bite. After realizing the bite, the patient found the crushed arthropod in her clothes in the right region around the navel. Examination revealed a localized edematous erythema with a necrotic lesion in the center measuring 1 cm in diameter, evoking the point of a spider bite, covered with numerous non-follicular sterile pustules with annular disposition (Figure 1). There was no other skin or systemic anomaly. Laboratory examination was normal. Histology objectified a spongiform pustule associated with an inflammatory infiltrate (Figure 2). The diagnosis of acute localized exanthematous pustulosis (ALEP) induced by a spider bite was retained.^1,2^ After a watchful waiting during 2 weeks, a complete regression was obtained. No relapse was noticed several months after.

Spiders are widely distributed over all continents. They can accidentally come into contact with humans. In North Africa, spiders are often harmless for humans and only a few species are likely to cause, as defensive behavior when threatened, real local or general reactions. Skin poisoning can cause acute generalized exanthematic pustulosis (AGEP) within a few hours, which has long been linked to a medicinal origin.^3^ Several cases of AGEP following a spider bite have been reported in the literature.^4,5^ Acute localized exanthematous pustulosis (ALEP) is a rare localized variant of AGEP.^2^

To our knowledge, this is the first report of an ALEP induced by a spider bite and localized on an unusual topography. This report aims to point out to this rare triggering factor in Tunisia, where incidents by spider bites may be underestimated.
